# Tolerability of BRAF and MEK Inhibitors for Metastasized Melanoma after Intra-Class Switch: A Multicenter, Retrospective Study

**DOI:** 10.3390/cancers15051426

**Published:** 2023-02-23

**Authors:** Martin Salzmann, Alexander Wald, Henner Stege, Carmen Loquai, Lisa Zimmer, Kinan M. Hayani, Lucie Heinzerling, Ralf Gutzmer, Alexander H. Enk, Jessica C. Hassel

**Affiliations:** 1 Department of Dermatology and National Center for Tumor Diseases, University Hospital Heidelberg, 69120 Heidelberg, Germany; 2 Department of Dermatology, University Medical Center Mainz, 55131 Mainz, Germany; 3 Department of Dermatology, Gesundheit-Nord Hospital Bremen, 28325 Bremen, Germany; 4 Department of Dermatology, University Hospital Essen, 45147 Essen, Germany; 5 Department of Dermatology, University Hospital Munich (LMU), 80336 Munich, Germany; 6 Department of Dermatology, University Hospital Erlangen, Friedrich Alexander University Erlangen-Nürnberg (FAU), 91054 Erlangen, Germany; 7 Skin Cancer Center, Department of Dermatology, Hannover Medical School, 30625 Hannover, Germany; 8 Department of Dermatology, Johannes Wesling Medical Center, Ruhr University Bochum, 32429 Minden, Germany

**Keywords:** melanoma, targeted therapy, BRAF inhibitor, toxicity

## Abstract

**Simple Summary:**

Patients suffering from metastasized melanoma can be treated with three different combinations of tablets (“targeted therapy”), with similar efficacy but different side effect profiles. When one of the combinations is not tolerated well, their physicians may switch to a different combination; however, it has not been evaluated whether the second combination is actually tolerated better. In this work, we collected data on 94 patients from six German cancer centers who received two different combinations of targeted melanoma therapy to figure out whether the second combination was tolerated better. We found that indeed many side effects did not occur again and the novel combination was overall tolerated better, which justifies switching the combination when a patient experiences severe side effects. However, new side effects may occur. We believe our results are important for oncologists to adequately counsel patients with poor tolerability of this commonly used melanoma treatment.

**Abstract:**

Targeted therapy with BRAF and MEK inhibitors (BRAFi, MEKi) is one of the mainstays of melanoma treatment. When dose-limiting toxicity (DLT) is observed, an option represents the intra-class switch to a different BRAFi+MEKi combination. Currently, there is scarce evidence for this procedure. This is a multicenter, retrospective analysis from six German skin cancer centers of patients who received two different combinations of BRAFi and MEKi. In total, 94 patients were included: 38 patients (40%) were re-exposed with a different combination because of previous unacceptable toxicity, 51 (54%) were re-exposed after progression, and 5 (5%) were included for other reasons. Of the 44 patients with a DLT during their first BRAFi+MEKi combination, only five (11%) experienced the same DLT during their second combination. A new DLT was experienced by 13 patients (30%). Six patients (14%) had to discontinue the second BRAFi treatment due to its toxicity. Compound-specific adverse events were avoided in the majority of patients by switching to a different combination. Efficacy data were similar to historical cohorts of BRAFi+MEKi rechallenge, with an overall response rate of 31% for patients who had previously progressed to treatment. We conclude that switching to a different BRAFi+MEKi combination if dose-limiting toxicity occurs is a feasible and rational approach in patients with metastatic melanoma.

## 1. Introduction

Treatment of metastatic melanoma (MM) has changed dramatically within the past decade. In addition to immune checkpoint inhibitors (ICI), targeted treatment (TT) with a combination of BRAF and MEK inhibitors (BRAFi+MEKi) is one of the mainstays of therapy in patients with BRAF-mutated MM. Currently, the combination of dabrafenib and trametinib (D+T) is the only approved BRAFi+MEKi combination in the adjuvant setting [1,2], while three combinations are approved for metastatic or unresectable melanoma: vemurafenib + cobimetinib (V+C) [3,4], D+T [5,6,7], and encorafenib + binimetinib (E+B) [8,9]. Since the efficacy of the different treatment options was similar in the respective phase 3 trials, the indication for each combination hinges on the specific toxicity profile and application schemes.

The adverse event (AE) profiles differ between the three combinations [10], with V+C commonly being associated with rash and photosensitivity, but also higher rates of gastrointestinal and hepatic toxicity, while D+T more frequently induces fever. E+B has a higher frequency of changes in blood count and facial paresis. Other AEs are similar among all combinations, such as arthralgia [11]. All combinations may have severe side effects; dose reduction or interruption was necessary in up to 50% of patients in all combinations, and about 15% of patients had to discontinue treatment permanently in the respective trials due to AE [10]. In these patients, a common pragmatic approach to overcome intolerance to one BRAFi+MEKi treatment is switching to a different BRAFi+MEKi combination. For example, a patient experiencing a severe cutaneous reaction during V+C may not experience this AE during D+T or E+B treatment. Furthermore, patients who were previously treated with ICI and may have ongoing high transaminases due to immune-related hepatitis might be best in a combination with a low frequency of changes in liver enzymes. However, to the best of our knowledge, there are currently only a few case reports [12,13,14] and no studies available that investigate AEs in patients receiving different consecutive BRAFi+MEKi combinations. Comparative studies of the differences in tolerability of the three combinations, especially within a single patient, are not available.

In addition to AE, another common reason for discontinuation of BRAFi+MEKi therapy is progressive disease, which may occur as primary resistance but mostly as acquired resistance over the course of treatment. Several reports and studies showed benefits of re-challenging TT after an interim treatment [15,16,17,18,19,20,21]. As a result, re-challenging of TT is a common approach. In clinical practice, this re-challenge may be initiated with the same or a different BRAFi+MEKi combination [22]. In a retrospective study by Tietze et al. on BRAFi+MEKi re-exposure, the majority of patients were treated with a different BRAFi+MEKi combination [16]. However, in that study, no data on differences in toxicity were reported.

The aim of our study is to investigate differences in toxicity, and report treatment efficacy after re-challenging patients with an intra-class switch of BRAFi+MEKi.

## 2. Materials and Methods

### 2.1. Patient Population

This is a multicenter, retrospective study. Patients were included if they received two different BRAFi+MEKi combinations for MM between 2014 and 2021. Patients were eligible if BRAFi+MEKi treatment was initiated either in metastatic disease (*n* = 87) or an adjuvant setting (*n* = 7). All patients received TT in routine clinical practice. The clinical data were collected from six German skin cancer centers. Data were extracted from patient records at the respective institutions. Anonymized data were merged centrally for analysis.

### 2.2. Treatment

Patients treated with any approved combination of BRAFi and MEKi were eligible. These included V+C at a dose of 1920 mg/60 mg per day, D+T at a dose of 300 mg/2 mg per day, and E+B at a dose of 450 mg/90 mg per day. Patients were treated according to the treatment standards of each institution until disease progression or unacceptable toxicity. Adverse Events (AE) were treated as clinically indicated and retrospectively graded according to the Common Terminology Criteria for Adverse Events (CTCAE) version 5.0 [23]. Dose-limiting toxicity (DLT) was defined as AEs requiring either permanent discontinuation of the drug, drug interruption, or permanent dose reduction.

### 2.3. Data Collection

Data on age, sex, details on the disease prior to initiation of therapy, previous treatment lines, the type of BRAFi+MEKi used for each combination, reasons for discontinuation, subsequent courses of disease and treatments, as well as efficacy data including best response, date of progression, and death/last contact, were collected. All treatment-related AEs were documented with the respective CTCAE grade and how the AE was managed. Documented laboratory values included LDH and S100 levels at each treatment initiation for their prognostic value [24,25].

### 2.4. Statistical Analyses

Statistical analysis was performed using IBM SPSS Statistics, version 27. The overall response rate (ORR) was defined as the percentage of patients achieving a partial response (PR) or complete response (CR) as the best response to therapy. Progressive disease (PD) was defined by disease recurrence at any site during observation according to standard RECIST criteria. The disease control rate (DCR) was defined as the proportion of patients with either a stable disease (SD), a PR, or a CR. Progression-free survival (PFS) was calculated from the initiation of each BRAFi+MEKi treatment until progression, OS was calculated from the initiation of the first BRAFi+MEKi treatment until death according to stage of disease. In patients with no events of progression or death at the time of final data analysis as well as in patients lost to follow-up, the date of last contact was used for censored calculations. Survival was estimated by the Kaplan–Meier method. Univariate comparisons of Kaplan–Meier estimators were performed using the log-rank test. For comparisons between groups with categorical variables, a two-sided Fisher’s exact and Chi-square test were used. Student’s *t*-test and Mann-Whitney-U tests were used to evaluate differences in LDH and S100 levels. *p* values were considered significant with values of *p* < 0.05.

## 3. Results

### 3.1. Patient Characteristics

A total of 94 patients were included, 48 males (51%) and 46 females (49%), with a median age of 56 years (range 19–85). Ten patients (11%) were treated in an adjuvant setting, of whom seven (7%) were treated in adjuvant stage III and three (3%) in adjuvant stage IV; 84 patients (89%) were treated for metastatic disease. The first BRAFi+MEKi combination was initiated as the first systemic treatment in 43 patients (46%), while the others received previous systemic treatment, which included immune checkpoint inhibitors in 38 patients (19 patients with anti-PD1 + anti-CTLA4-inhibitor and 19 with anti-PD1 monotherapy, respectively), adjuvant interferon-α in eight patients, BRAFi monotherapy in four patients, and dacarbazine in one patient.

Kaplan-Meier estimated the median OS of stage IV patients after initiation of the first BRAFi+MEKi combination at 46.9 months (95% confidence interval [CI]: 29.2–64.6 months); the median OS of adjuvant patients was not reached. Fifty-six patients (62%, 8/10 [80%] of adjuvant patients) were alive at the time of data collection.

Patient characteristics are summarized in Table 1.

### 3.2. Treatment Characteristics

As their first BRAFi+MEKi combination, 23 patients (25%) received V+C, 65 patients (69%) received D+T, and six patients (6%) received E+B. Of the 94 patients, 51 (54%) received a second BRAFi+MEKi combination as re-exposure after progression on the first combination, 38 (40%) to overcome previously experienced toxicity, and five (5%) due to other reasons, which included the approval of a different combination in four cases (while the first combination had been administered as an off-label use) and difficulty swallowing large pills in one case.

Fifty-two patients (55%) received an interim treatment between the two BRAFi+MEKi combinations, of which 47 received a PD1-inhibitor: 12 patients as monotherapy, 25 patients combined with a CTLA4-inhibitor, and ten patients in combination with other systemic agents; three patients received anti-CTLA4 treatment as monotherapy, and two patients received chemotherapy. Forty-two patients (45%) received no systemic interim treatment. In this collective, the median time between initiation of the first BRAFi+MEKi and initiation of the second BRAFi+MEKi combination was 3.1 months (range 0.6–59.1 months). Thirty-one of these 42 patients (74%) were switched to a different combination due to adverse events.

**Figure 1:**
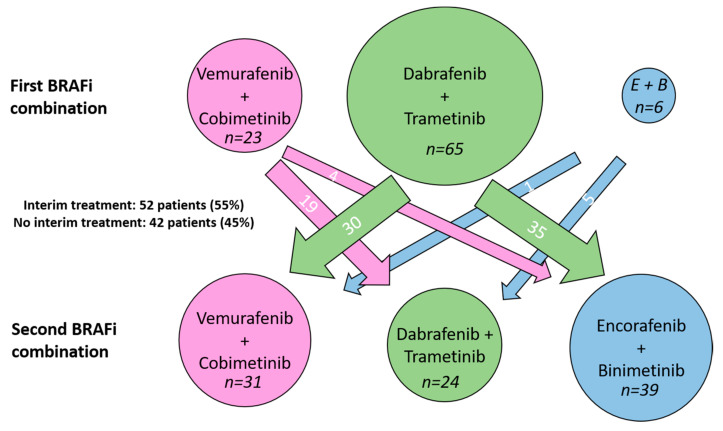
Number of patients treated with each BRAF+MEK inhibitor combination and the respective switch to the new combination treatment.

As their second BRAFi+MEKi combination, 31 patients (33%) received V+C, 24 patients (26%) received D+T, and 39 patients (42%) received E+B. Figure 1 illustrates the number of patients who switched between the different combinations.

Of the 38 patients who discontinued the first BRAFi+MEKi combination due to toxicity, 11 received V+C, 26 received D+T, and one received E+B. For their second BRAFi+MEKi combination, 12 received V+C, 11 received D+T, and 15 received E+B.

### 3.3. Adverse Events

Of the 94 patients, 44 (47%) experienced a DLT during their first BRAFi+MEKi treatment. In 30 of these, discontinuation of the first BRAFi+MEKi treatment was necessary. In another 8 patients, discontinuation was not deemed necessary by the investigators, but a second BRAFi+MEKi combination was initiated to reduce ongoing AE with the aim of increasing patients’ quality of life. The highest-grade AE was grade 3 CTCAE in 36 patients (38%), grade 2 in 20 patients (21%), and grade 1 in 19 patients (20%). In 19 patients (20%), no AE was reported.

During the second BRAFi+MEKi combination, 33 patients (35%) experienced a DLT, 29 of these patients did not experience the same DLT during the first combination. The highest-grade AE was grade 3 CTCAE in 30 patients (32%), grade 2 in 22 (23%), and grade 1 in 16 (17%). 26 patients (28%) reported no AE. Of the 33 patients with DLT during the second BRAFi+MEKi combination, 17 (52%) already experienced some DLT during the first BRAFi+MEKi combination. Of the 50 patients with no DLT during their first BRAFi treatment, 16 (32%) experienced a new DLT.

**Figure 2:**
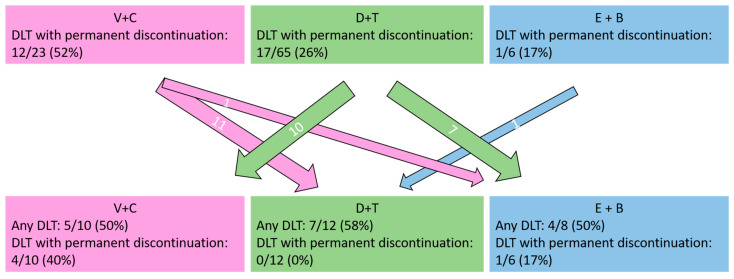
Number of patients treated with the respective BRAF+MEK inhibitor combinations (vemurafenib + cobimetinib [V+C], dabrafenib + trametinib [D+T], encorafenib + binimetinib [E+B]); the first combination was discontinued due to dose-limiting toxicity (DLT). Numbers in the arrows represent the number of patients switching to the respective second BRAFi+MEKi combination.

A summary of all reported AE is given in Table 2. Figure 2 shows a summary of the 30 patients who discontinued their first BRAFi+MEKi combination due to dose-limiting toxicity with their respective treatment switch and the tolerability of the second BRAFi+MEKi combination.

#### 3.3.1. Patients with a Second BRAFi+MEKi Combination Due to Adverse Events

Overall, 44 patients experienced a DLT during the first combination; in 38 of these patients, investigators considered DLT to be the main reason to switch the BRAFi+MEKi combination. Of the 44 patients who experienced a DLT during the first combination, only five (11%) experienced the same DLT again (kidney failure in two patients, rash, arthralgia, and gastrointestinal AEs in each one). The most commonly overcome DLTs included rash in six patients (three were switched away from V+C, and three from D+T), pyrexia in five patients (two were switched away from V+C, and three from D+T), and gastrointestinal AEs in three patients (all were switched away from D+T). Of the 44 patients who experienced a DLT during the first BRAFi+MEKi combination, 27 (61%) experienced no DLT, while 17 (39%) experienced a DLT, of which six (14%) had to permanently discontinue the second BRAFi+MEKi combination as well; only one of these was due to the same previously experienced toxicity (arthralgia). Overall, we observed a reduction in the highest grade-AE in 25 out of 44 patients (57%). Thirteen patients (30%) developed new DLTs different from the previously experienced ones, of which one patient developed a new DLT in addition to the recurrence of a previously experienced DLT. An overview of the number of patients experiencing DLT during the second BRAFi+MEKi combination is given in Table 3.

#### 3.3.2. Compound-Specific Toxic Effects

**Figure 3:**
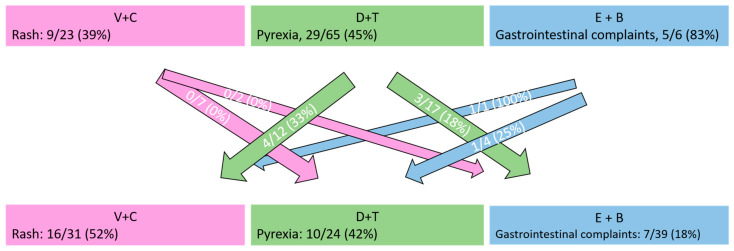
Number of patients suffering from class-specific side effects during the respective BRAF+MEK inhibitor combination (vemurafenib + cobimetinib [V+C], dabrafenib + trametinib [D+T], or encorafenib + binimetinib [E+B]). Numbers in arrows represent the number of patients who experienced the same side effect during the second BRAFi+MEKi combination as well (e.g., 0/7 patients with a rash during V+C also experienced a rash during D+T).

Two common compound-specific toxic events include rash for V+C and pyrexia for D+T. Nine of 23 patients (39%) in our collective developed drug-induced rash during V+C as their first BRAFi+MEKi treatment, with six patients developing grade 3 rash. None of these nine patients developed any rash during subsequent D+T and E+B treatments. Similarly, pyrexia occurred in 29 out of 65 patients (45%) during the first treatment with D+T, consisting of ten cases with grade 1, 13 cases with grade 2, and six cases with grade 3. Only seven of these 29 patients (24%) developed pyrexia during subsequent V+C or E+B treatment, two of which were grade 3. Only one of the six patients receiving E+B as their first BRAFi+MEKi combination was switched due to toxicity, which was kidney failure. The most common, lower-grade toxicity in these patients was gastrointestinal. Class-specific side effects occurred in both treatment lines, as shown by Table 2 and Figure 3. Figure 3 illustrates class-specific side effects with their respective occurrences during the first and second BRAFi+MEKi combinations.

#### 3.3.3. Patients with the Second BRAFi+MEKi Combination after Progressive Disease with the First Combination

Fifty-one patients received a second BRAFi+MEKi combination as re-exposure after experiencing progressive disease with the first combination. Thirteen of these (25%) already had a DLT during the first BRAFi+MEKi combination. During the second BRAFi+MEKi combination, 16 patients (31%) developed a DLT, with 15 experiencing different or new toxic effects. Seven patients (14%) had to permanently discontinue the second BRAFi+MEKi treatment due to adverse events.

### 3.4. Treatment Efficacy

Treatment efficacy is only reported for patients treated in the metastatic, non-adjuvant setting. The ORR of the first BRAFi+MEKi combination was 58% (41/71, 23 not evaluable) and the DCR was 86% (61/71). The median PFS was 7.8 months (95% CI: 5.7–9.9 months). The ORR of the second BRAFi+MEKi combination was 51% (47/92, 2 not evaluable) and the DCR was 67% (63/92), hence lower compared to the first BRAFi+MEKi treatment. Similarly, the median PFS of the second BRAFi+MEKi combination was lower at 4.9 months (95% CI: 4.0–5.8 months).

Patients who discontinued BRAFi+MEKi, due to unacceptable toxicity, showed a very favorable outcome when introduced to the novel BRAFi+MEKi combination, with an ORR of 83% (30/36, 2 not evaluable) and a DCR of 97% (35/36), with ten CRs observed. The median PFS of the second BRAFi+MEKi combination was 13.8 months (95% CI: 2.7–24.9 months) in these patients; within this collective, patients who discontinued their first BRAFi+MEKi due to adverse events without an event of progression and without interim treatment showed a median PFS of 21.5 months (95% CI: 19.0–24.0 months) from the initiation of their first BRAFi treatment (*n* = 26). Patients with a DLT during their first combination did not show a significantly prolonged OS compared to patients without a DLT (median not reached vs. 38.7 months [95% CI: 20.7–56.7 months], *p* = 0.248). Patients who received a second BRAFi+MEKi combination after progression to the previous BRAF+MEKi treatment showed significantly worse efficacy; however, the ORR at re-exposition was 31% (16/51, *p* < 0.001) and the DCR 51% (26/51, *p* < 0.001). Patients with progression to the first treatment showed a trend towards shorter PFS, which was not statistically significant (4.5 months [95% CI: 2.4–6.6 months] vs. 7 months [95% CI: 1.6–12.4 months], *p* = 0.116).

The groups of patients treated with the respective BRAFi+MEKi combinations were homogeneous in terms of treatment situation, LDH, and S100 levels; thus, no comparative statistics between the different BRAFi+MEKi combinations are reported in this manuscript.

## 4. Discussion

The main aim of our study was to assess intra-individual differences in the toxicity of BRAFi+MEKi combinations in melanoma patients after an intra-class switch. To the best of our knowledge, no previous studies have investigated whether an intra-class switch may result in improved tolerability in patients who discontinued targeted therapy due to AE, and no such studies are currently available.

Our data show that in the majority of patients experiencing dose-limiting AE, switching to a different BRAFi+MEKi combination can effectively overcome the toxicity. We observed better tolerability of the second combination, as only 11% of our patients experienced the same DLT again and only 16% had to discontinue the second BRAFi+MEKi combination. This accounts especially for the two most common drug-dependent AEs: V+C-induced rash did not occur in any of our patients when we introduced a different combination, and D+T-induced pyrexia was only apparent in 24% of patients during the second combination. Thus, based on our small cohort, we recommend switching to a different BRAFi+MEKi combination when dose-limiting side effects occur, especially if these are compound-specific.

However, it has to be noted that the overall incidence of AEs was only slightly reduced. Previous studies suggested a generally lower toxicity rate during BRAFi+MEKi re-challenge [17], which our study cannot confirm after an intra-class switch. While most previously experienced DLTs did not occur again, 31% of our patients developed a new DLT when treated with a different BRAFi+MEKi combination. While previous DLTs can be effectively avoided, the occurrence of previously unknown toxicity was frequent in our patients.

This has to be considered, especially when re-treating patients with a different combination after they developed resistance to the previous BRAFi+MEKi. Based on our small patient collective, switching to a different combination may trigger new toxic effects with potentially reduced tolerability in patients with no DLT during their first BRAFi+MEKi combination. This occurred in 32% of our patients. Our efficacy data with an ORR of 31% and a DCR of 51% in patients retreated after progression to the first BRAFi+MEKi combination is similar to the previous analysis by Cybulska-Stopa et al. [17], whose study population of 51 patients had previously progressed to treatment. Studies by Tietze et al. [16] and Valipone et al. [18] support the approach of re-challenge; however, patients receiving BRAFi monotherapy were eligible. In neither of the studies, a population who received the same BRAFi (with or without MEKi) during rechallenge compared to a population with an intra-class switch was clearly defined. Thus, with a lack of a historical control group, clear evidence for the potential superiority of an intra-class switch vs. re-introduction of the same BRAFi+MEKi combination during a re-challenge is still elusive and cannot be provided by our data herein. In fact, based on our findings on toxicity, the potential occurrence of new dose-limiting AEs should always be taken into consideration and may even speak against an intra-class switch in patients with good tolerability of the first combination. In order to provide evidence on the potential superiority of an intra-class switch, a prospective randomized trial of patients receiving the same BRAFi+MEKi combination vs. patients receiving an intra-class switch would be necessary. In all retrospective patient cohorts of the mentioned studies, as well as in our collective, efficacy data should be evaluated with care due to the inhomogeneous patient characteristics. Nevertheless, the exceptionally favorable outcome of patients who received a second BRAFi+MEKi combination due to the toxicity of the first combination is noticeable. When patients did not progress to their first treatment and received no interim treatment, the median PFS from initiation of BRAFi+MEKi to progression during the second combination of 21.5 months is far superior to that reported in historical cohorts of BRAFi+MEKi treatment [4,5,6,7,8,9]. The phenomenon of improved treatment efficacy of BRAFi+MEKi treatment in the presence of side effects, especially of an inflammatory nature, has been suggested previously [11,26]. Our efficacy data may further encourage physicians to switch BRAFi+MEKi combinations when dose-limiting side effects occur; still, the small collective with many potential confounders does not allow definitive conclusions on efficacy, Thus, each patient’s case should be evaluated individually when making a treatment decision.

It has to be noted that the strategy of switching combinations is off-label in the adjuvant setting, with only the D+T combination being approved.

We are aware of the many limitations of this study. These mainly include the retrospective design, a small number of patients, as well as an inhomogeneous disease status and treatment intention. Still, we believe we must provide enough data to justify an intra-class switch in patients with DLT during BRAFi+MEKi treatment and enable treating physicians to better counsel patients.

## 5. Conclusions

If dose-limiting toxicity occurs, switching to a different BRAFi+MEKi combination is feasible, as most DLTs are not re-induced after the intra-class switch. This accounts especially for compound-specific DLTs. However, new adverse events occurred.

## Figures and Tables

**Table 1:** Patient characteristics. Serum levels of lactate dehydrogenase and S100 are given for the initiation of the first BRAFi+MEKi combination.

	Stage IV *n* = 87	Adjuvant Stage III *n* = 7
Gender		
Male	43 (49%)	5 (71%)
Female	44 (51%)	2 (29%)
Age	Median 55 years (range 19–85)	Median 62 years (range 43–83)
Previous systemic treatment lines		
None	37 (43%)	6 (86%)
PD1 + CTLA4-inhibitors	19 (22%)	
PD1-inhibitor monotherapy	18 (21%)	1 (14%)
Interferon α	8 (9%)	
BRAF inhibitor monotherapy	4 (5%)	
Dacarbazine	1 (1%)	
Serum lactate dehydrogenase		
Normal	34/72 (47%)	5 (71%)
Elevated	38/72 (53%)	2 (29%)
S100 serum levels		
Normal	27/68 (40%)	5 (71%)
Elevated < 2 × ULN	16/68 (24%)	
Elevated ≥ 2 ×ULN	25/68 (37%)	2 (29%)

**Table 2:** All reported adverse events (AE) with their respective grades according to the CTCAE. Several AEs were possible in the same patient. Other * adverse events included: photosensitivity, pericardial effusion, arterial hypertension, loss of appetite, hyponatremia, hypercholesterinemia, acute kidney failure, alopecia, myalgia, sunburn, glomerulonephritis, erythema nodosum, neutropenia with erysipelas, sinusitis, hemophagocytic lymphohistiocytosis, elevated lipase and alcalic phosphatase, pancreatitis, hypophosphatemia, dyspnea, lymphopenia, and ageusia.

Adverse Event (AE)	First BRAFi+MEKi Combination																		Second BRAFi+MEKi Combination																	
	V+C (*n* = 23)						D+T (*n* = 65)						E+B (*n* = 6)						V+C (*n* = 31)						D+T (*n* = 24)						E+B (*n* = 39)					
	Grade 1		Grade 2		Grade 3		Grade 1		Grade 2		Grade 3		Grade 1		Grade 2		Grade 3		Grade 1		Grade 2		Grade 3		Grade 1		Grade 2		Grade 3		Grade 1		Grade 2		Grade 3	
	*n*	%	*n*	%	*n*	%	*n*	%	*n*	%	*n*	%	*n*	%	*n*	%	*n*	%	*n*	%	*n*	%	*n*	%	*n*	%	*n*	%	*n*	%	*n*	%	*n*	%	*n*	%
Highest grade AE	4	17	6	26	7	30	9	14	17	26	23	35	4	67	1	17	1	17	6	19	9	29	11	36	5	21	7	30	6	25	5	13	6	15	13	33
Pyrexia	3	13	1	4	1	4	10	15	13	20	6	9	0	0	1	17	0	0	3	10	2	6	1	3	2	8	5	21	3	13	0	0	2	5	1	3
Rash	2	9	1	4	6	26	6	9	2	3	3	5	2	33	0	0	0	0	5	16	3	10	8	26	0	0	0	0	0	0	3	8	0	0	1	3
Fatigue	1	4	1	4	0	0	8	12	3	5	3	5	1	17	0	0	0	0	2	6	1	3	0	0	2	8	1	4	0	0	4	10	1	3	0	0
Gastrointestinal	1	4	2	9	0	0	10	15	3	5	5	8	5	83	0	0	0	0	3	10	3	10	1	3	1	4	3	13	0	0	1	3	2	5	4	10
Elevated liver enzymes	2	9	0	0	1	4	0	0	2	3	1	2	0	0	0	0	0	0	0	0	0	0	0	0	1	4	0	0	1	4	0	0	1	3	3	8
Panniculitis	0	0	0	0	0	0	0	0	3	5	1	2	0	0	0	0	0	0	0	0	0	0	0	0	0	0	1	4	0	0	0	0	0	0	0	0
Cardiac toxicity	0	0	2	9	0	0	0	0	2	3	1	2	0	0	0	0	0	0	0	0	0	0	0	0	0	0	0	0	1	4	0	0	0	0	0	0
Elevated creatine kinase	0	0	1	4	1	4	4	6	2	3	2	3	1	17	0	0	0	0	0	0	1	3	0	0	0	0	0	0	0	0	0	0	1	3	0	0
Arthralgia	1	4	0	0	1	4	7	11	5	8	2	3	0	0	0	0	0	0	1	3	3	10	1	3	1	4	1	4	1	4	0	0	2	5	1	3
Pulmonal toxicity	0	0	0	0	0	0	0	0	1	2	2	3	0	0	0	0	0	0	0	0	1	3	0	0	0	0	1	4	0	0	0	0	0	0	0	0
Ocular toxicity	1	4	0	0	0	0	0	0	0	0	1	2	0	0	0	0	0	0	*0*	0	1	3	1	3	0	0	0	0	0	0	0	0	0	0	3	8
Peripheral edema	0	0	0	0	0	0	0	0	1	2	0	0	1	17	0	0	0	0	0	0	0	0	0	0	0	0	0	0	0	0	0	0	0	0	0	0
Thrombopenia	0	0	0	0	0	0	0	0	1	2	0	0	0	0	0	0	0	0	0	0	0	0	0	0	0	0	0	0	0	0	0	0	0	0	0	0
Other *	2	9	3	13	6	26	8	12	3	5	2	3	1	17	0	0	1	17	3	10	0	0	1	3	*0*	0	0	0	2	8	2	5	3	8	3	8

**Table 3:** Summary of dose-limiting toxicities (DLT) occurring during the second BRAFi+MEKi combination in patients with or without previous DLT during the first combination. One patient had a recurrent case of the same DLT as well as a new DLT.

Patients with Dose-Limiting Toxic Effects (DLT) during 1st BRAFi+MEKi Combination	*n* = 44
DLT also occuring during the second BRAFi+MEKi combination	17 (39%)
The same DLT occurred again	5 (11%)
New DLT	13 (30%)
No DLT during the second BRAFi+MEKi combination	27 (61%)
Patients without DLT during the 1st BRAFi+MEKi combination	N = 50
New DLT during the second BRAFi+MEKi combination	16 (32%)

## Data Availability

The datasets used and/or analyzed during the current study are available from the corresponding author on reasonable request.
